# The Use of the Schizonticidal Agent Quinine Sulfate to Prevent Pond Crashes for Algal-Biofuel Production

**DOI:** 10.3390/ijms161126035

**Published:** 2015-11-17

**Authors:** Chunyan Xu, Kangyan Wu, Steve W. Van Ginkel, Thomas Igou, Hwa Jong Lee, Aditya Bhargava, Rachel Johnston, Terry Snell, Yongsheng Chen

**Affiliations:** 1 School of Civil and Environmental Engineering, Georgia Institute of Technology, 200 Bobby Dodd Way, Atlanta, GA 30313, USA; xuchunyan92@gatech.edu (C.X.); kk.wu@gatech.edu (K.W.); steven.van.ginkel@ce.gatech.edu (S.W.V.G.); thomas.igou@gatech.edu (T.I.); hlee702@gatech.edu (H.J.L.); abhargava38@gatech.edu (A.B.); 2 School of Biology, Georgia Institute of Technology, 201 Cherry-Emerson, Atlanta, GA 30313, USA; terry.snell@biology.gatech.edu (R.J.); rachel.johnston@biology.gatech.edu (T.S.)

**Keywords:** biodiesel production, algae pond crash, toxicity, quinine sulfate, rotifer

## Abstract

Algal biofuels are investigated as a promising alternative to petroleum fuel sources to satisfy transportation demand. Despite the high growth rate of algae, predation by rotifers, ciliates, golden algae, and other predators will cause an algae in open ponds to crash. In this study, *Chlorella kessleri* was used as a model alga and the freshwater rotifer, *Brachionus calyciflorus*, as a model predator. The goal of this study was to test the selective toxicity of the chemical, quinine sulfate (QS), on both the alga and the rotifer in order to fully inhibit the rotifer while minimizing its impact on algal growth. The QS LC_50_ for *B*. *calyciflorus* was 17 µM while *C. kessleri* growth was not inhibited at concentrations <25 µM. In co-culture, complete inhibition of rotifers was observed when the QS concentration was 7.7 µM, while algal growth was not affected. QS applications to produce 1 million gallons of biodiesel in one year are estimated to be $0.04/gallon or ~1% of Bioenergy Technologies Office’s (BETO) projected cost of $5/gge (gallon gasoline equivalent). This provides algae farmers an important tool to manage grazing predators in algae mass cultures and avoid pond crashes.

## 1. Introduction

Algae are considered a promising source of biofuel for the future [1] due to high energy prices [2], the use of non-agricultural land for algal production [3], high algal areal productivity [4], potential net greenhouse-gas (GHG) emissions benefits [5], and potential use of wastewater as a nutrient source and CO_2_ from power plants [6,7]. However, in open pond systems, algae are preyed upon by higher organisms such as rotifers, ciliates, other algae, *etc.* which must be controlled in order for algae biofuel’s true potential to be realized.

Among the common small (<200 um) invertebrates, rotifers are recognized as a vital component of aquatic ecosystems [8]. According to an article written by Montemzzani *et al.* (2015) [9], rotifers grow faster than ciliates and can form large population densities—1000 to 500,000 individuals/L in highly eutrophic environments. Rotifers perform the most rapid reproduction of metazoan zooplankton and can reproduce asexually with doubling times less than a day under circumstances with high nutrients, neutral pH, and high temperature [9]. Due to all these reasons, it is vital to control algal predators if algae are needed to produce biofuels.

*B. calyciflorus* belongs to the animal kingdom, lives in freshwater environments, and is an ideal test organism because of its global distribution, sensitivity, easy cultivation, and short generation time [10]. *B*. *calyciflorus* can eat thousands of algae cells and cause a pond crash within days [11]. As it is difficult to remove rotifers via mechanical methods, chemical treatment is suggested as an alternative solution [9]. Preferred chemicals are selectively toxic to rotifers and not as toxic to algae. In order to inhibit predation, chemicals can be introduced to algae ponds which are selectively toxic to predators while not affecting the growth of algae.

In this study, quinine sulfate (QS) was chosen as the chemical, *B*. *calyciflorus* was chosen as the model predator, and *Chlorella kessleri* was chosen as the model alga. QS is a cinchona alkaloid, which is very basic and usually present as a salt. This alkaloid used to be extracted from cinchona tree bark but now all quinine is synthesized [12]. QS is an anti-protozoan agent and was first used to target the protozoan parasite, species of *Plasmodium* (*Plasmodium* sp.), which causes malaria. QS is effective in curing malaria as it can inhibit the growth of *Plasmodium* sp. by inhibiting glycolysis, as well as the synthesis of protein and nucleic acid. It creates hemozoin which decreases the detoxification of heme [13]. It kills the schizont—acting on the erythrocyte stage of *Plasmodium* sp. QS is considered a schizonticidal agent because it targets organisms that reproduce by schizogony or asexually [14]. Rotifers, a type of algal predators, reproduce asexually via diploid, ameiotic parthenogenesis [12], and thus the hypothesis of this study is that QS is selectively toxic to rotifers at concentrations not affecting algae growth. The QS may also inhibit mitochondrial ATP-regulated potassium channels which may not be present in algae [13].

In previous toxicity studies, the concentration of toxicant, temperature, pH, and fluid motion were tested [15,16]. In the study of controlling predator ciliates of *Dunaliella salina*, the result showed that QS can eliminate ciliates while not affecting the algae [17]. The 72 h EC_50_ for algae was 14.5 mg/L, while the 24 h LC_100_ for the ciliate was 12–14 mg/L. An algae pond resisted the attack from ciliates and recovered when a dose of 10 mg/L QS was added. QS is able to block potassium channel and disrupt osmotic regulation in ciliates. Species of *Dunaliella* (*Dunaliella* sp.), can survive the presence of QS as it has a high percentage of organic osmolytes to resist the effect of blocked potassium channels [15]. In the study investigating how fluid motion modifies pentachlorophenol (PCP) toxicity to *B. calyciflorus*, the LC_50_ of *B. calyciflorus* decreased from 738 µg/L in static conditions to 262 µg/L in fluid motion which suggests QS would work better in more mixed ponds [16].

## 2. Results and Discussion

**Figure 1:**
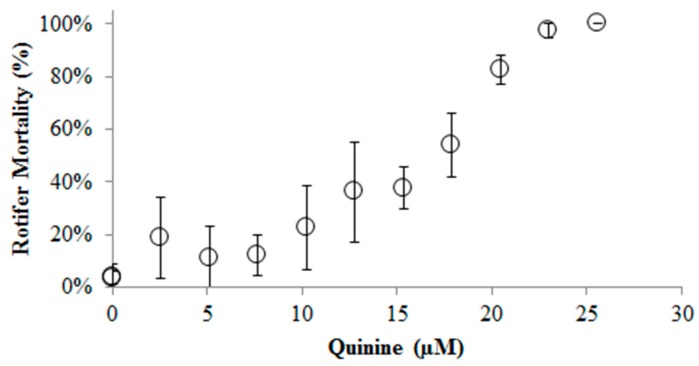
Effect of quinine sulfate on *B*. *calyciflorus*. (Circles stand for the rotifer mortality at different quinine sulfate concentrations).

**Figure 2:**
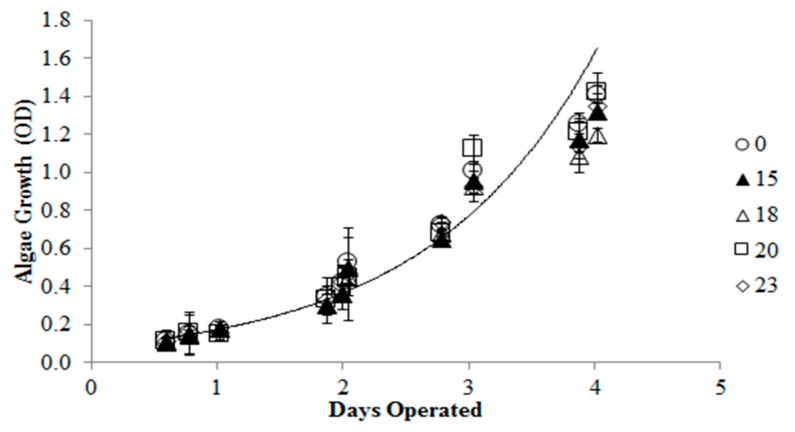
Effect of quinine sulfate (0, 15, 18, 20, 23 uM) on *C*. *kessleri*.

This experiment consisted of three parts. The first part tested QS toxicity on *B. calyciflorus* (Figure 1). As QS increased, rotifer mortality increased. The 24 h QS LC_50_ on *B. calyciflorus* was approximately 17.4 µM (14 mg/L) and 100% mortality was observed at concentrations above 23 µM. The second part tested QS toxicity on *C*. *kessleri* (Figure 2). According to the LC_50_ obtained for the rotifer, QS concentrations of 0, 15, 18, 20, and 23 uM were tested in 100 mL algae shake flask suspensions. QS at concentrations up to 23 µM did not have any effect on the growth of *C*. *kessleri*.

**Figure 3:**
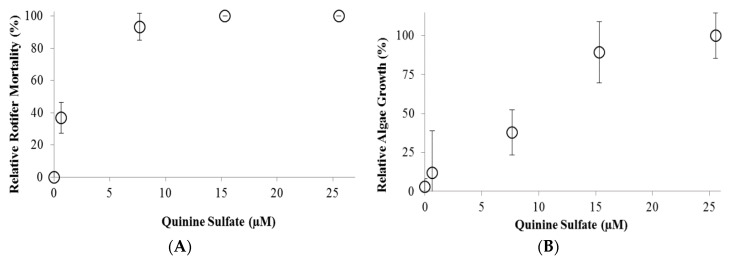
Effect of quinine sulfate (QS) on a co-culture of *C. kessleri* and *B*. *calyciflorus*. (**A**) Effect of quinine sulfate on *B*. *calyciflorus*. (Circles stand for the rotifer mortality at different quinine sulfate concentrations); (**B**) Effect of quinine sulfate on *C. kessleri*. (Circles stand for the algae growth rates at different quinine sulfate concentrations).

**Figure 4:**
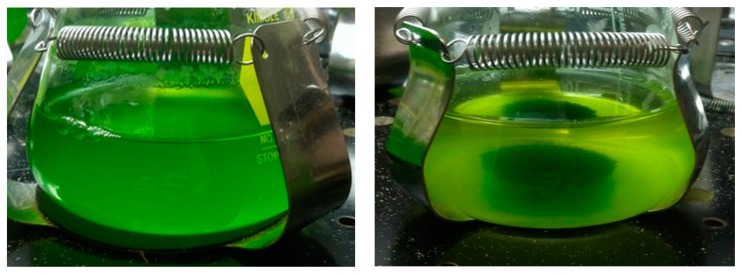
Before (**left**) and after (**right**) photos of a *C. kessleri* pond crash by *B*. *calyciflorus*.

The third part tested QS toxicity on a co-culture of *C. kessleri* and *B*. *calyciflorus* at QS concentrations of 0, 0.6, 7.7, 15, and 26 µM. On day seven, when control rotifers reached their peak population, the relative rotifer mortality rate of the treatments were calculated (Figure 3). At QS concentrations above 7.7 µM, rotifer mortality approached 100% while algae growth did not differ from the positive controls. On average, the algae growth rate (an increase of 0.37 OD units/day) at the highest QS concentration of 25 µM did not differ from the controls (an increase of 0.38 OD units/day). QS concentrations lower than the rotifer LC_50_ are effective in co-cultures because only rotifer reproduction needs to be inhibited—immediate rotifer mortality is not necessary if reproduction is inhibited [18]. At QS concentrations less than 7.7 µM, QS did not inhibit the rotifers and predation reduced algae growth to <50% of the positive controls causing the flasks to eventually crash (*i.e.*, the algae flocculated and a solid mass of rotifers, digested algae, and coagulated algae developed in the center of the flask separate from the clear supernatant (Figure 4). On day 7, rotifer numbers reached approximately 14,000, 9000, and 1000 rotifers/100 mL at QC concentrations of 0, 0.6, and 7.7 µM, respectively.

The persistence of QS over 10 days was confirmed by re-inoculating the 26 µM QS experiment with newly hatched rotifers. 100% rotifer mortality was observed after 24 h which shows that QS remains toxic to rotifers for at least 10 days in solution.

In order to produce 1 million gallons of biodiesel in a year, we calculate a QS cost of $0.04/gallon biodiesel or ~1% of Bioenergy Technologies Office’s projected cost of $5/gallon gasoline equivalent (gge) by 2019. This assumes a QS cost of $1/kg, an algae production rate of 20 g·m^−2^·day^−1^, 50% lipid content, a biodiesel density of 880 kg/m^3^, and a QS application of 7.7 µM which persists for two weeks (26 doses per year) [16,19].

## 3. Experimental Section

The freshwater alga *C. kessleri* was obtained from the University of Texas at Austin algae collection (UTEX #2228). The freshwater rotifer *B. calyciflorus* was originally collected in Gainesville, Florida [20]. BG-11 was used as the algae growth media [21]. The optical density (OD) of algal suspensions was obtained at 750 nm using a Thermo Spectronic Genesys 20 spectrophotometer (Fisher Scientific, Pittsburgh, PA, USA). Rotifer eggs hatched after 16 h in petri dishes containing ~40 mL of spring water in a 25 °C Barnstead Lab-line 305 Imperial III incubator (Barnstead International, Dubuque, IA, USA) with continuous light (40 W) with a vertical light path of ~20.3 cm. Rotifers were counted using a stereomicroscope (SMZ-2T, Nikon Co., Tokyo, Japan).

### 3.1. Acute QS (Quinine Sulfate) Toxicity Tests on B. calyciflorus

Snell *et al.* [15,22] standardized toxicity tests with freshwater rotifers. Three experiments were conducted with four replicates at five different QS concentrations ranging from 0 to 25.5 µM in 24-well plates at 1 mL per well. Wells were covered to prevent evaporation. Ten to twelve rotifers were transferred into each well and were incubated for 24 h. The well plates were examined under the stereomicroscope for rotifer mortality after 24 h to calculate LC_50_s (the LC_50_ is the concentration that causes mortality to 50% of the population). The number of live and dead rotifers was recorded, with rotifers not moving for 10 s regarded as dead.

### 3.2. QS Toxicity Tests on C. kessleri

Stock cultures of *C. kessleri* were grown in 800 mL glass columns (5.1 cm wide and 64 cm tall) with a conical bottom, were given continuous illumination from the side from twelve 40-Watt 1.2 m long cool white fluorescent light bulbs with a light path of approximately 7.6 cm, and sparged with air at ~100 mL/min. Log-phase algal suspensions (100 mL) were then used to initiate 250 mL Erlenmeyer shake flask batch experiments in which different amounts of QS were added. The shake flask experiments were illuminated from above with a light path of ~48 cm. The temperature was 23 °C and the mixing speed was 150 rpm (Platform Shaker: Innova 2100, New Brunswick Scientific, Enfield, CT, USA).

Based on the LC_50_ obtained from the acute rotifer toxicity tests, algae suspensions were spiked with QS concentrations of 0, 15, 18, 20, and 23 µM in order to determine the algae EC_50_, the effective concentration that inhibits algae growth by 50%. Initial algae OD was 0.1 in the QS toxicity tests on *C. kessleri* experiments. Tests were conducted in triplicate. OD was measured daily between 0 and 96 h to determine algal growth rate as a function of QS concentration.

### 3.3. QS Toxicity Tests on a Co-Culture of B. calyciflorus and C. kessleri

In order to represent an algae pond susceptible to predation, the rotifer and alga were co-cultured to determine the toxic effect of QS on *B. calyciflorus* in the presence of algae. The same setup as in Section 3.2 was used, except rotifers were added to the algae cultures. Two experiments were conducted with different QS concentrations (0.6, 7.7, 15.3 and 25.5 µM) in triplicate. Initial rotifer densities averaged six rotifers per mL. One positive control with algae, rotifers, and no QS was conducted in triplicate. The initial OD of the algae was 0.2. Rotifers were counted each day over 8 days. Rotifers were counted in 1 mL samples in a well plate under the stereomicroscope. If the number of rotifers per 1 mL sample was greater than 40, the field of view of the well plate was divided into four quarters and each quarter was counted and multiplied by four to get the rotifer number per 1 mL sample in the co-cultures; this rotifer count multiplied by 100 is rotifer number in the flask. If the rotifer count in each quarter exceeded 40, a smaller sample size of 0.2 mL was used and the number of rotifers was then multiplied by 500 to obtain the rotifer number in in each flask. The relative mortality of the rotifers and the growth of algae were calculated using the positive controls when the rotifer concentrations were at their peak according to: Abs (1− (rotifer # in the experiment/positive control rotifer #)) × 100 (see Equation (1)), which # stands for the count of rotifer number. The standard deviation is the average standard deviation of each experiment/positive control rotifer # × 100 (see Equation (2)).

(1) $$\left|1-\frac{\#\text{ of rotifers in the experiment}}{\#\text{ of rotifers in the positive control}}\right|\;\times \;100$$

(2) $$\frac{\text{average standard deviation of each experimental run}}{\#\text{ of rotifers in the positive control}}\;\times \;100$$

The relative growth of the alga at each QS concentration is the average growth rate/growth rate at the highest concentration of QS × 100 (see Equation (3)). The standard deviations are of the replicate relative growth rates of the alga.

(3) $$\frac{\text{average growth rate at a QS concentration}}{\text{growth rate at the highest QS concentration}}\;\times \;100$$

### 3.4. Testing the Persistence of QS

The persistence of quinine sulfate in the environment is not well documented although it is said to be stable under normal temperatures and pressures and may decompose when exposed to light [15]. In order to confirm QS persistence, the co-culture experiment with 25.5 µM QS was re-inoculated after 10 days with newly hatched rotifers.

## 4. Conclusions

*B. calyciflorus* is more sensitive to QS than *C*. *kessleri* with a rotifer LC_50_ of 17.4 µM. In co-culture, after seven days of QS exposure, a QS concentration greater than 7.7 µM was adequate to reduce predation low enough to allow *C. kessleri* to grow at its maximum rate. Recycling of the algae harvest water would retain QS in a closed looped system which would reduce application costs and alleviate any discharge impacts on the environment. The data and calculations from these experiments aid in the development of algal biofuels and QS may be a useful tool to help algae farmers protect their crop.
